# Ecological niche models reveal divergent habitat use of Pallas's cat in the Eurasian cold steppes

**DOI:** 10.1002/ece3.9624

**Published:** 2022-12-14

**Authors:** Niloufar Lorestani, Mahmoud‐Reza Hemami, Azita Rezvani, Mohsen Ahmadi

**Affiliations:** ^1^ Department of Natural Resources Isfahan University of Technology Isfahan Iran

**Keywords:** clades, climate change, conservation planning, habitat suitability

## Abstract

Identifying the association between the patterns of niche occupation and phylogenetic relationships among sister clades and assisting conservation planning implications are of the most important applications of species distribution models (SDMs). However, most studies have been carried out regardless of within taxon genetic differentiation and the potential of local adaptation occurring within the species level. The Pallas's cat (*Otocolobus manul*) is a less‐studied species with unknown biogeography and phylogenetic structure across a widespread yet isolated range from the Caucasus to eastern China. In the current study, by considering a previously proposed genetic structure and based on a cluster analysis on climatic variables, we supposed three clades for this species, including *O. m. manul*, *O. m. ferrugineus*, and *O. m. nigripectus*. We developed SDM for each clade separately and compared it with a general distribution model of the species to determine whether the hypothesized taxonomic resolution affects the predicted ecological niche of the within‐species structures. We assessed the effect of climate change on the future distribution of the species to detect the most sensitive clades to global warming scenarios. Our results showed that for all clades' models, the AUC and TSS were greater than the general model. Access to the preferred prey of the Pallas's cat, that is, pika, had a significant effect on the distribution of *O. m. manul* and *O. m. ferrugineus*, whereas the most influential variable affecting *O. m. nigripectus* habitat suitability was terrain slope. Based on our future projections, we found that future climate change likely threatens the clades *O. m. ferrugineus* and *O. m. nigripectus* more than *O. m. manul*, findings that were hidden in the general model. Our results highlight the proficiency of SDMs in recognizing within‐taxon habitat use of widespread species and the necessity of this procedure for implementing effective conservation planning of these species.

## Graphical abstract

The Pallas's cat with a widespread but isolated distribution shows a divergent habitat selection across Eurasian cold steppes. A sub‐taxon climate change projection revealed the different responses of the Pallas's cat subspecies to future global warming.

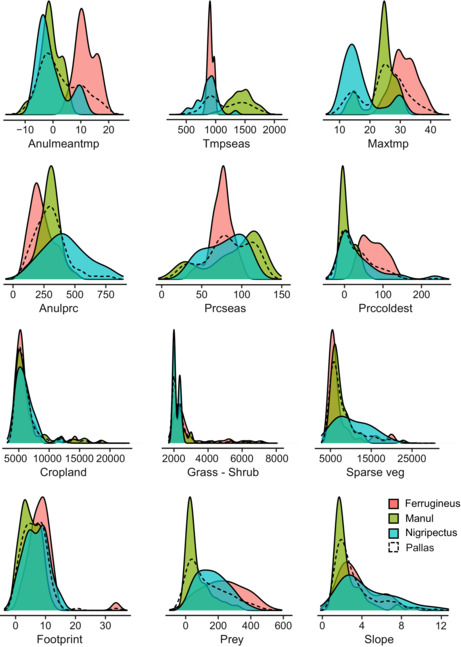

## 1 INTRODUCTION

Over the last decades, impressive advances in species distribution models (SDMs) have led to their widespread use as a promising tool in conservation planning and biodiversity management (Guisan et al., 2013). Specifically, assessing the distribution of the species with declining populations due to human threats or environmental change is one of the operative applications of SDMs (Thorn et al., 2009). Species distribution models by combining data of species occurrence and environmental variables predict suitable habitats that can provide the crucial requirements of species survival (Elith et al., 2011; Guisan et al., 2013; Merow et al., 2013). SDMs have widely been used for identifying species' suitable habitats and assessing their niche partitioning (Franklin, 2010; Hemami et al., 2018), evaluating the distribution of species in broad geographic scales (Brito et al., 2009), prioritizing the conservation planning and gap analysis (Ahmadi et al., 2020; Carvalho et al., 2011), and predicting the impact of climate change on the species distribution (Khosravi et al., 2021; Parmesan, 2006; Rocca & Milanesi, 2022; Salas et al., 2018; Shabani et al., 2019).

In particular, investigating the association between the patterns of niche occupation and phylogenetic relationships of sister taxa (Ahmadi et al., 2018; Morales‐Castilla et al., 2017), and identifying cryptic speciation to assist phylogenetic comparison (Ahmadzadeh et al., 2013; Gutiérrez‐Tapia & Palma, 2016) are supportive applications of SDMs. Although combining SDMs with genetic analysis could allow for conservation planning implications (Habel et al., 2011; Ikeda et al., 2017; Marcer et al., 2016), most SDM‐based studies still do not consider the genetic differentiation and varying potentials of local adaptation within the species level.

In many SDMs studies, species presence data are collected from various databases, from datasets that are obtained in direct field surveys to those compiled from existing databases such as Global Biodiversity Information Facility (GBIF), museums, and atlases. Despite gaining more information on species distribution, this might lead to modeling inefficiency (Sillero & Barbosa, 2021). For instance, clumping occurrence points might lead to spatial autocorrelation and subsequent overestimation in favor of denser areas (Dormann et al., 2007). Yet, several methods have been recommended to deal with this issue, for example, spatial filtering and background data manipulation (Kanagaraj et al., 2013; Phillips et al., 2009). Nevertheless, there might be a pattern of within‐species niche variation in many cases, especially for those across broad geographical scales. It is generally believed that local adaptation leads to the occupation of a wide range of environmental conditions, particularly for those with a wide distribution range (Cushman et al., 2014). This theory suggests that the magnitude of local adaptation positively correlates with within‐population genetic divergence and, accordingly, with environmental and phenotypic differentiation (García‐Ramos & Kirkpatrick, 1997). In this case, grouping occurrence points of different clades in one dataset may result in inflation in the predicted niche of the species. This is due to the fact that there is a positive relationship between range size and niche breadth (Moore et al., 2018); hence, aggregating within‐taxon structures may result in smoothed response curves across environmental gradients (Pearman et al., 2010), which in turn leads to inflate niche breadth and overestimate predicted distribution of the target taxa.

In the current research, we focused on modeling the habitat suitability of Pallas's cat (*Otocolobus manul*) across its global distribution range for two reasons: first, it has a widespread distribution, and second, due to its specialized habitat selection, there is a possibility of divergent niche structures in this species (Kitchener et al., 2017). Supporting this idea, the occurrence of distinct phylogenetic structures for this species has been proven; nevertheless, the level of differentiation is yet to be confirmed. Pallas's cat is a small cat of the subfamily Felinae, which is currently listed as least concern by the IUCN (Ross et al., 2020). The widespread distribution of this species covers a wide variety of environmental conditions from northeastern China throughout Central Asia to the Caucasus and from the Himalayas to the southern borders of the Taiga forests (Moqanaki et al., 2019). Across this extended range, the remnant populations of the species are limited to small and isolated habitat patches that are becoming increasingly fragmented, and probably locally extinct, due to habitat destruction (Ross et al., 2016). Human development, fatality due to anthropogenic disturbance, predation by herding dogs, depletion of preferred prey, and specialized social behavior and habitat selection are among the main threats that are intensifying the decline of the Pallas's cat populations (Farhadinia et al., 2016; Greco et al., 2022; Joolaee et al., 2014; Ross, Barashkova, et al., 2019; Ross, Moqanaki, et al., 2019). While these known threats act primarily at local scales, a further large scale but unknown concern is climate change which threats the long‐term persistence of the Pallas's cat across its entire range.

Overall, climate change has become a major threat to biodiversity globally due to its widespread impacts. Climate change generally affects the functioning and structure of ecosystems (Pio et al., 2014; Smeraldo et al., 2021). Specifically, climate change impacts geographic distribution and population dynamics at the species level and forces mismatch in biological interactions at a community level (Garcia et al., 2014). It may move species' suitable habitats outside the boundaries of protected areas or areas with more human activity, which can subsequently increase conflict between humans and species (Wilson et al., 2017). Species respond differently to climate change depending on the severity and velocity of changing factors (Loarie et al., 2009) and on within‐species genetic diversity (Chen et al., 2010; Evans et al., 2016). Most studies have analyzed the effects of climate change on biodiversity at the species level (Parmesan, 2006; Pearson et al., 2006; Root et al., 2003), and the effects in within‐species diversity have been less investigated (Habel et al., 2011). This is in spite of the fact that preserving genetic diversity under drastic and rapid global change is one of the top priorities in conservation biology (Pauls et al., 2013).

Generally, climate change‐induced range shifts in the species distribution occur in two patterns: latitudinal range shift and altitudinal range contraction (Loarie et al., 2009; Parmesan, 2006; Trivedi et al., 2008). Species with limited distribution and dispersal abilities as well as mountain‐dwelling ones are among the most sensitive groups to climate change (Parmesan, 2006; Pearson & Dawson, 2003). Rangwala et al. (2013) showed that mountains heat up faster than lowland areas. Moreover, species in upper trophic levels, for example, carnivores, are more sensitive to climate change (Voigt et al., 2003). Accordingly, as a predatory species with widespread but fragmented distribution in Eurasian mountain steppes, the Pallas's cat is likely one of the most sensitive species to climate change.

Taxonomically, the Pallas's cat is the only species in the genus *Otocolobus* and shares a common ancestor with Asian leopard cats of the genus *Prionailurus* (Li et al., 2016). Phylogenetically, three clades of the Pallas's cat have been described: (i) *O. m. manul* (hereafter Manul) introduced by Pallas (1776) and occurring in Russia, Mongolia, Kazakhstan, and southern Siberia (Altai, Tuva, Transbaikalia); (ii) *O. m. nigripectus* (hereafter Nigripectus) identified by Hodgson (1842) and distributing in Tibet, Kashmir, Nepal, and Bhutan; and (iii) *O. m. ferrugineus* (hereafter Ferrugineus) described by Ognev (1928) and occupying Iran, Afghanistan, Pakistan, Turkmenistan, Uzbekistan, and Tajikistan (Kitchener et al., 2017). Kitchener et al. (2017) argued that Ferrugineus is equivalent to Manul due to the lack of genetic investigations. Nonetheless, wide distribution with various climatic and habitat conditions can raise the possibility of a distinct and cryptic within‐species structure in the Pallas's cat.

Generally, despite its broad distribution, the habitat selection pattern and the environmental factors affecting the Pallas's cat are poorly understood. Although the habitat use of the species has been recently investigated at local (Greco et al., 2022) and broad (Greenspan & Giordano, 2021) geographical scales, the lack of scientific understanding of the within‐taxon status of the species restricts the development of effective conservation planning measures. Therefore, this study was conducted with the primary purpose of investigating habitat suitability and identifying the factors affecting the species distribution throughout the Eurasian cold steppes. We also assumed three distinct groups in Pallas's cat global distribution based on a within‐species clustering analysis on their occupied climatic niche. We then performed SDMs for each group separately. By doing so, we were interested in determining whether the hypothesized taxonomic resolution affects the predicted ecological niche of within‐species structures. Accordingly, we compared clades model with the general model of the species to investigate whether our approach accounts for probable inflation in the predicted niche of the species. Finally, as divergent within‐taxa structures respond to climate change differently (Pearman et al., 2010), we adopted our approach to climate change projections to model the species' future distribution and to find the most sensitive clade to future global warming scenarios.

## 2 MATERIALS AND METHODS

### 2.1 Study area

**FIGURE 1:**
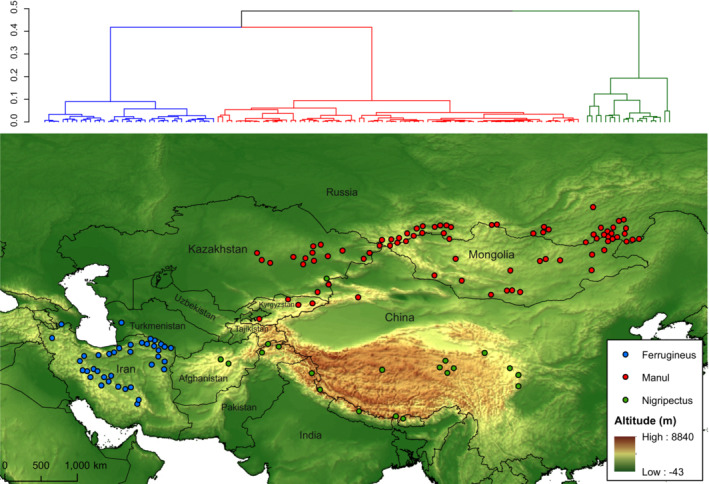
Dendrogram (top) and geographic distribution of Pallas's cat climatic clusters (down) resulting from an agglomerative hierarchical clustering of climatic variables at the occurrence points based on a Euclidean dissimilarity matrix.

The study area covers the entire global distribution of the Pallas's cat from the Caucasus to eastern China (Figure 1). Overall, we chose the study area in a way that embraces all the potential habitats of the species, including the Caucasus, Iranian Plateau, Hindu Kush, Himalayas, Tibetan Plateau, Altai‐Sayan region, and South Siberian Mountains. This region is characterized by an outstanding topographic diversity extending from the arid deserts of Iran and Central Asia to the massive mountain ranges of the Himalayas and Tibetan Plateau. For example, Werhahn et al. (2018) found that Pallas's cats in the Himalayan region disperse in areas with an altitude range of 450–5593 m. The Pallas's cat mainly inhabits rocky mountain steppes and semi‐desert foothills in this region (Ross et al., 2020). However, due to its specialization in habitat preference, the remaining populations of the species are patchily distributed across the region (Chalani et al., 2008; Dibadj et al., 2018; Farhadinia et al., 2016; Joolaee et al., 2014; Talebi Otaghvar et al., 2017). From a climatic viewpoint, the region is largely characterized by continentality, a large variation in annual temperature and aridity.

### 2.2 Species data

The occurrence points of the species were compiled from three resources. First, we downloaded the relevant data of the species presence from the Global Biodiversity Information Facility (GBIF, 2021a, 2021b, 2021c, 2021d, 2021e, 2021f) and filtered out occurrence points with inaccurate spatial information using the package “CoordinateCleaner” (Zizka et al., 2019) in the R environment. Second, we reviewed the relevant scientific literature on the distribution of the species across its entire range (details of the occurrence points of the literature review per country are presented in Appendix S1: Table S1). To ensure the accuracy of the occurrence points obtained from the GBIF dataset, we visually checked them with those compiled from the literature review in ArcGIS. Third, we conducted a field survey and compiled direct observations recorded by game guards of the Department of Environment (DoE) in the Iranian range of the species. Before the SDM analysis, to reduce negative effects of the probable spatial autocorrelation of the presence points (Dormann et al., 2007), duplicate points in a 5‐km radius were removed, nearly equal to the grid size of the SDM analysis. Accordingly, 137 presence points (38 points for Ferrugineus, 19 points for Nigripectus, and 80 points for Manul) remained for the SDM analysis.

The SDM analysis was performed based on the existence of ecologically distinct groups in the Pallas's cat distribution. To identify these groups, we applied a hierarchical cluster analysis on the climatic characteristics of the species occurrence points, and based on the obtained dendrogram, they were assigned to the identified clusters. We first downloaded 19 climatic rasters from the WorldClim dataset (Hijmans et al., 2005). Next, we calculated the Euclidean dissimilarity matrix of 19 climatic variables at the species occurrence points by using the package “vegan” in the R environment. To identify climatic clusters, we then used the agglomerative hierarchical clustering with the function *agnes* of the package “cluster” as this method provides the best performance in the biogeographical regionalization analysis (Kreft & Jetz, 2010). We based this intraspecific climatic differentiation for a cluster‐specific SDM analysis.

### 2.3 Predictor variables

In this study, we prepared environmental variables for two modeling approaches; to predict the current habitat suitability of Pallas's cat and to evaluate possible changes in the species distribution under climate change scenarios. To investigate the habitat suitability of Pallas cat based on reviewing previous studies (Farhadinia et al., 2016; Ross, 2016; Ross et al., 2020), five categories of environmental factors were selected, including land cover, climatic, anthropogenic, topographic, and prey availability.

For land cover, we used the Land Cover map produced by Climate Change Initiative (CCI) of the European Space Agency (ESA) for the year 2015 (ESA, 2017). With a pixel size of 300 m, this dataset includes 37 land cover classes. To avoid increasing the number of input variables, we recoded similar cover types that are of the same ecological functioning and focused on three important land cover types for the species, including croplands, the mixture of grass/scrub/shrub lands, and sparse vegetation. To use them as continuous variables in the SDM analysis, we extracted these three concatenated cover type and calculated Euclidean Distance from each cover types using the ArcMap v.10.5. Climatic variables were obtained from the WorldClim database at 2.5 arc‐minutes (~ 4 km) resolution (Hijmans et al., 2005). We used the variance inflation factor (VIF) with “usdm” package (Naimi, 2017) in R environment to evaluate multicollinearity among climatic predictors. We excluded variables with VIF > 6 and six climatic variables remained for the SDM analysis including annual mean temperature, temperature seasonality, maximum temperature of warmest month, annual precipitation, precipitation seasonality, and precipitation of coldest quarter.

To quantify anthropogenic effects in our modeling approach, we used the human footprint raster layer developed by Venter et al. (2018). This variable is created by combining data on population density and the presence of human infrastructure (e.g., road networks and human access). We used the digital elevation model (DEM) of the Shuttle Radar Topography Mission (SRTM) (http://srtm.csi.cgiar.org) and generated the slope raster layer as a measure of topographic variability in the SDM analysis. All variables were resampled to the pixel size of climatic variables (Table 1).

**TABLE 1:** Details of the eco‐geographic factors used for SDM analysis

Category	Variable	Abbreviation	Unit	Source
Land cover	Sparse vegetation	Sparse veg	‐	ESA CCI Land Cover
	Grass/scrub/shrub land	Grass—Shrub	‐	
	Crop land	Cropland	‐	
Anthropogenic	Human footprint	Footprint	‐	Venter et al. (2018)
Topographic	Slope	Slope	Degree	SRTM's DEM
Biological	Pika habitat suitability	Prey	‐	This study
Climatic	Annual mean temperature	Anulmeantmp	°C	WorldClim
	Temperature Seasonality (standard deviation × 100)	Tempseas	°C	
	Max Temperature of Warmest Month	Maxtemp	°C	
	Annual Precipitation	Anulprc	mm	
	Precipitation Seasonality	Prcseas	mm	
	Precipitation of Coldest Quarter	Prccold	mm	

Pika is the primary prey for the Pallas's cat across the entire range of the species (Adibi et al., 2018; Fox & Dorji, 2007; Webb et al., 2014). The Pallas's cat highly prefers this species because it is two to four times larger than other small mammals' preys, and hence, optimizes energy intake per unit foraging of the Pallas's cat (Ross et al., 2012, 2020). In order to incorporate the role of prey availability in the habitat suitability of Pallas's cat, we also generated pika's habitat suitability map with the same selected layers for Pallas's cat suitability model (details of the pika's habitat suitability modeling are provided in Appendix S1: Figure S2). The VIF of all predictors were checked again to assess their multicollinearity. As no variable with a VIF greater than 6 was seen, we used all of them in the SDM analysis.

### 2.4 SDM analysis

The species distribution modeling was performed for the global distribution and a within‐species clustering approach separately. For each modeling attempt, we used one regression‐based method—generalized linear models (GLM), and three machine learning algorithms—generalized boosting model (GBM), random forest (RF), and maximum entropy (MaxEnt), with the package biomod2 (Thuiller et al., 2009) in R v 4.1. Results of the models were then combined into an ensemble model to take into account the degree of uncertainty specific to each method in an integrated model. We generated a random sample of 10,000 background points within a buffer of 250 km radius around occurrence points. We used this buffer area to avoid selecting background points from inaccessible regions that may cause model overestimation. For each model, we used a spatially blocked cross‐validation procedure using the package block‐CV (Valavi et al., 2019) in the R environment to split presence and background points into training and test datasets. Using this procedure avoids error underestimation in the spatial predictions and provides a robust method for estimating the predictive performance of the models (Roberts et al., 2017). The performance of the models was evaluated using the area under the curve (AUC) of a receiver operating characteristic (ROC) plot (Phillips et al., 2006) and the true skill statistic (TSS) (Allouche et al., 2006). The final ensemble model was calculated based on the weighted average of the four models considering their AUC and TSS values. To compare the results of general and individual clades' modeling, for each clade, we computed the extent of suitable habitats and the percent of overlapped habitats identified in each modeling approach. To do this, we first converted the continuous suitability maps to binary suitability maps based on the 10th percentile training presence threshold. We used this threshold for two reasons: It allowed us to remove points in marginal areas with very low habitat conditions and to take into account the potential spatial bias or uncertainties caused by outlier occurrence points (Ahmadi et al., 2020).

### 2.5 Climate change modeling

To assess the impact of climate change on the future distribution of Pallas's cat and compare the effect of climate change on each clade separately, we used climatic‐only SDM analysis for the current time and 2050. Assessing climatic projections was performed based on four global circulation models (GCMs), including CCSM, GFDL‐CM3, Hedgem‐ES, and MIROC, and two representative concentration pathways (RCP), including RCP 2.6 and RCP 8.5. Similar to the species distribution models, we employed the before‐mentioned predictive methods and combined them into an ensemble model using the package biomod2 (Thuiller et al., 2009). For the climate change projections, all model parameterizations were implemented similar to the habitat suitability predictions. Using raster calculator analysis for each RCP scenario, we averaged the obtained climatic suitability maps of four GCMs.

We converted climatic ensemble maps to binary maps by using the 10th percentile training presence threshold. Usually, predictive models are not very reliable when projected to outside of the training domain (Elith et al., 2010). This is especially true for climate change predictions where the fitted model based on the current condition is projected to novel future climatic conditions. A good solution is to measure the similarity between the new environments (future climatic conditions) and those in the training sample (Elith et al., 2010) based on the multidimensional environmental similarity surface (MESS) analysis. Accordingly, in this study, after climate change projection, we also generated the MESS map and used negative MESS values, that is, dissimilar areas, to crop the future projections. Then, based on the binary maps of current and future projections and using the biomod2 package, two indices were calculated to show the proportion of range change; (i) habitat gain, meaning the number of pixels that are currently not occupied but predicted to be in the future, and (ii) habitat loss, indicating the number of pixels that are suitable in the current but predicted to be nonsuitable in the future.

## 3 RESULTS

### 3.1 Species distribution models

The hierarchical clustering dendrogram indicated three distinct climatic clusters for the Pallas's cat occurrence points (Figure 1). Accordingly, all occurrence points were distinctively resolved in the potential cluster, except for one point geographically belonged to the Manul clade but located in the Nigripectus clade. Based on geographic proximity, we assigned this point to the Manul clade. After implementing SDMs, the AUC and TSS values of models in the general model (without considering clades) were lower than those of clades' models (Table 2). Among the clades, Ferrugineus obtained the highest AUC (all models >0.9) and TSS (all models >0.8) values.

**TABLE 2:** The predictive performance of the general and clades species distribution models (values in the parenthesis indicate standard errors of 10 replication).

	GLM		GBM		RF		Maxent	
	AUC	TSS	AUC	TSS	AUC	TSS	AUC	TSS
General model	0.816 (0.031)	0.542 (0.061)	0.861 (0.025)	0.616 (0.055)	0.847 (0.014)	0.573 (0.024)	0.865 (0.021)	0.614 (0.046)
*O. m. ferrugineus*	0.921 (0.06)	0.824 (0.106)	0.955 (0.039)	0.879 (0.086)	0.928 (0.046)	0.816 (0.099)	0.97 (0.012)	0.866 (0.074)
*O. m. manul*	0.899 (0.017)	0.732 (0.051)	0.922 (0.012)	0.759 (0.03)	0.902 (0.031)	0.713 (0.061)	0.903 (0.018)	0.704 (0.052)
*O. m. nigripectus*	0.88 (0.07)	0.71 (0.146)	0.834 (0.088)	0.633 (0.105)	0.789 (0.083)	0.573 (0.166)	0.913 (0.021)	0.805 (0.058)

Based on the results, the order of the importance of the variables in the habitat suitability model of each clade was different (Table 3). For Manul, temperature seasonality, annual mean temperature, sparse vegetation, and maximum temperature of the warmest month were identified as the four most important variables, respectively. For Ferrugineus, prey, temperature seasonality, sparse vegetation, and annual mean temperature were the most important variables, respectively. For Nigripectus, temperature seasonality, prey, slope, and maximum temperature of the warmest month were the four most important variables, respectively (Table 3).

**TABLE 3:** Average relative importance of explanatory variables in the habitat suitability model (HSM) and climatic suitability model (CSM).

	Ferrugineus		Manul		Nigripectus	
	HSM	CSM	HSM	CSM	HSM	CSM
Anulmeantmp	0.221	0.458	0.376	0.332	0.199	0.455
Tempseas	0.333	0.516	0.367	0.715	0.288	0.16
Maxtemp	0.125	0.406	0.173	0.416	0.172	0.069
Anulprc	0.119	0.565	0.168	0.177	0.086	0.107
Prcseas	0.096	0.201	0.115	0.209	0.07	0.529
Prccold	0.152	0.691	0.099	0.063	0.103	0.396
Footprint	0.071		0.063		0.073	
Grass—Shrub	0.036		0.082		0.147	
Cropland	0.13		0.044		0.08	
Sparse veg.	0.239		0.314		0.185	
Prey	0.631		0.075		0.283	
Slope	0.073		0.151		0.202	

*Note*: The later was used in climatic change projections. For variables description, see Table 1.

**FIGURE 2:**
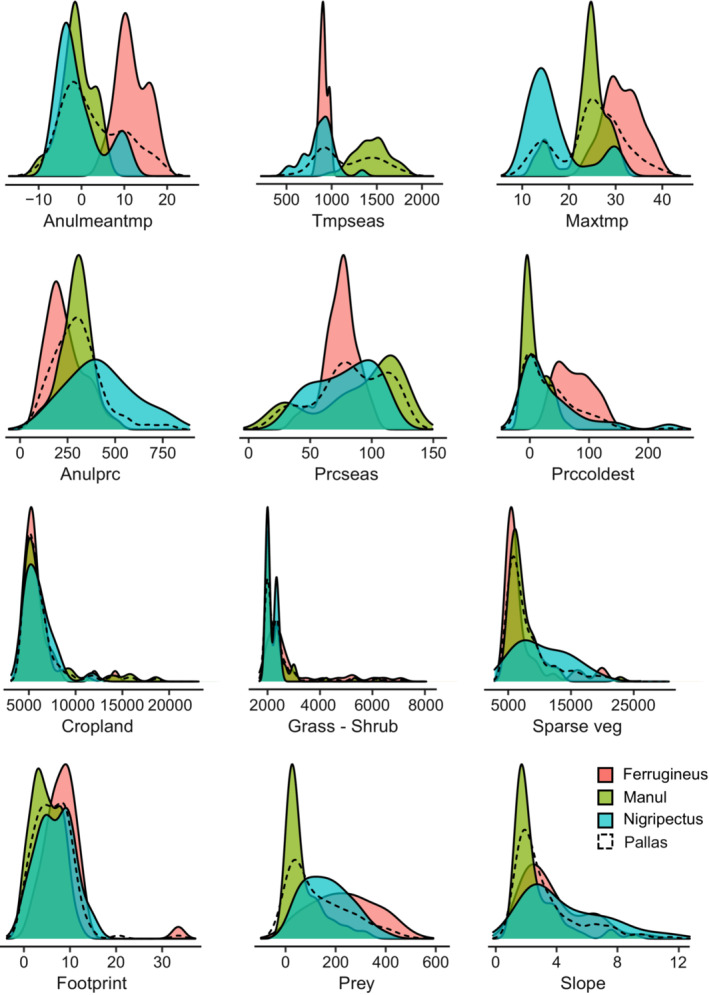
Habitat suitability map of Pallas's cat derived based on the general model (pooled set of the occurrence points of the clades) and individual clades' models. Habitat suitability of: (a) *O. m. manul*, (b) all Pallas's cat by general model, (c) *O. m. ferrugineus*, (d) *O. m. nigripectus.*

The most suitable areas for Ferrugineus clade were predicted in the mountainous regions of Alborz and Zagros in the north and west of Iran and limited parts of northwestern Pakistan and southeastern Afghanistan (Figure 2c). However, in the general model, many parts of eastern Iran and central Afghanistan were identified as suitable habitats (Figure 2b). For this clade, we also found that, with 50.43% spatial overlap, the extent of suitable habitats in the general model was greater than that of the individual clade model (Table 4; Appendix S1: Figure S1). Unlike Ferrugineus, the extent of Nigripectus suitable habitats in the general models was almost half of the clade model with the lowest percent of spatial overlap (24.7%, Table 4). For this clade, only limited areas over the mountains of Pamir and Karakorum between eastern China, Tajikistan, and northern India were predicted as suitable areas in the general model (Figure 2b). However, in its clade model, in addition to Pamir and Karakorum, extensive ranges of the Himalayas, the Yanggula, and Qilian mountains in the south and central China were also identified as suitable habitats (Figure 2d). Similarly, for Manul, the extent of suitable habitat in the general model was lower than that of the clade model, and comparing the two modeling approach, 65.6% of the suitable habitats were spatially overlapped (Table 4; Appendix S1: Figure S1). For this clade, the predicted suitable habitats were mostly stretched over mountains of central Asia from Tian Shan Mountain between China, Tajikistan, Kyrgyzstan, and Kazakhstan to Altay Mountain between Kazakhstan and Mongolia, and to cold steppes of northeastern Mongolia (Figure 2a).

**TABLE 4:** The extent of suitable habitats and the percentage of habitat overlap based on the general and clades models.

	General model (Km^2^)	Clades models (Km^2^)	Habitat overlap (%)
*O. m. ferrugineus*	627363.26	467200.08	50.43%
*O. m. nigripectus*	550417.46	1107198.70	24.7%
*O. m. manul*	2119148.06	2463826.94	65.6%

*Note*: A threshold of 10th percentile training presence was used to identify suitable habitats.

### 3.2 Climate change projections

**FIGURE 3:**
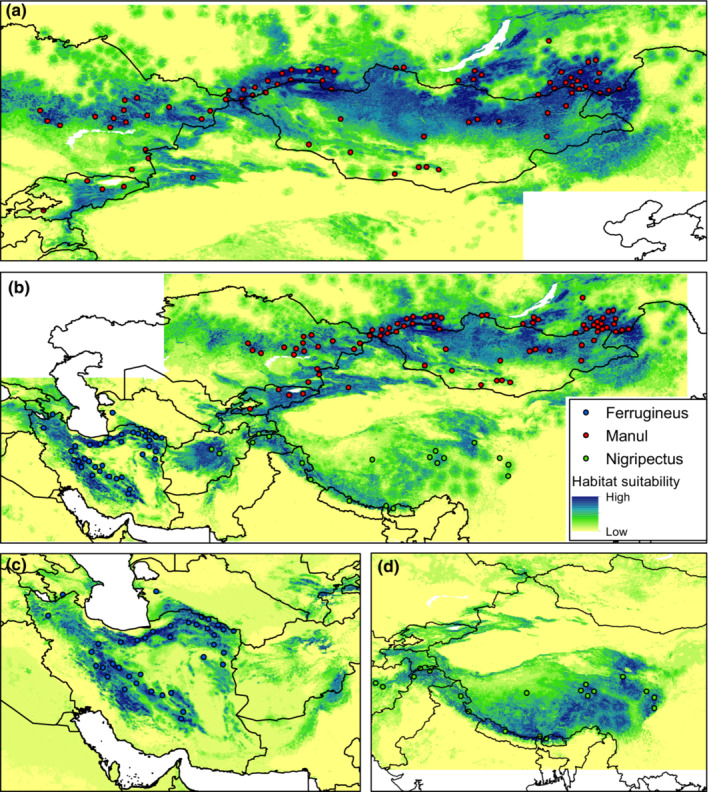
Climatic suitability models of the individual clades of Pallas's cat obtained for current time and projected to 2050. Dashed lines indicate dissimilar areas with negative MESS values.

**FIGURE 4:**
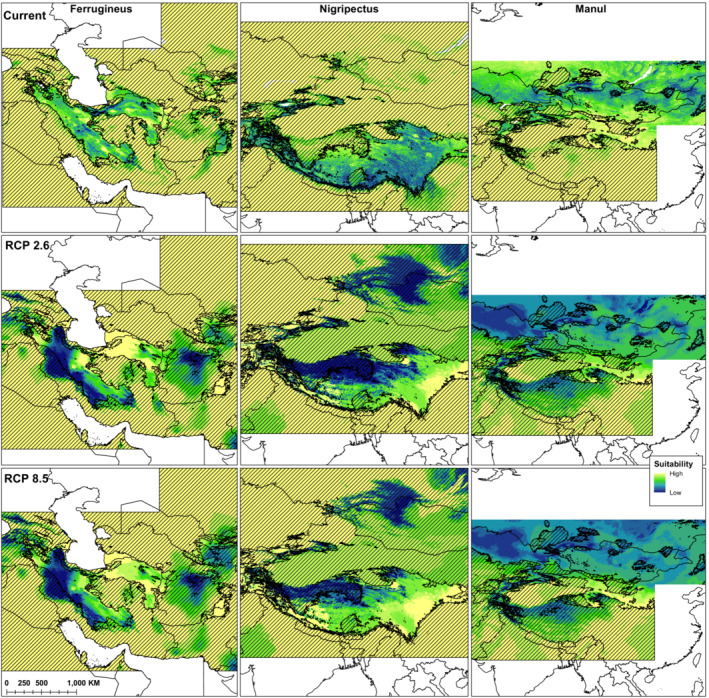
Mean and standard deviation of the habitat loss and habitat gain in the general (pooled set of the occurrence points of the clades) and individual clades’ models obtained based on climate change projections from current to 2050.

Similar to species distribution models, in climate change modeling, the AUC and TSS values of the general models were lower than when modeling was performed for each clade separately (Table 5). In this situation and among the clades, again the Ferrugineus model showed the highest predictive performance, of which MaxEnt had the highest AUC and TSS values (0.976 and 0.919, respectively). In climate change projections, like habitat suitability modeling, the three clades responded to climatic variables differently (Table 3). For Manul, temperature seasonality, maximum temperature of warmest month, and annual mean temperature were the three most important variables. Precipitation of coldest quarter, annual precipitation, and temperature seasonality were the most important predictors for Ferrugineus. For Nigripectus, precipitation seasonality, annual mean temperature, and precipitation of coldest quarter were the three most important climatic variables (Table 3). The climate change projections showed that habitat change in the general model differed significantly from the clade models (Figures 3 and 4). This pattern was more pronounced for Manul, for which the projection of current climate suitability in the general model to future climatic scenarios indicated habitat losses of 99.95% (RCP 2.6) and 99.98% (RCP 8.5) by 2050 (Figure 4). Whereas in the clade model, Manul was projected to lose less than 1% of its suitable habitat, and interestingly, the extent of its suitable habitats will increase (90% in RCP 2.6 and 86% in RCP 8.5). For Ferrugineus, habitat loss was projected to be 65% and 68% in RCP 2.6 and RCP 8.5, respectively, and habitat gains were projected as only 7% and 8%, respectively, by 2050. The greatest habitat loss among the clades was obtained for Nigripectus, for which habitat loss was projected to be 71% (RCP 2.6) and 88% (RCP 8.5) by 2050 (Figure 4). Also, the lowest habitat gain was obtained for this clade (2% and 3% under RCP 2.6 and RCP 8.5 climate change scenarios, respectively, Figure 4).

**TABLE 5:** The predictive performance of the general and clades' climate change models (values in the parenthesis indicate standard errors of 10 replication).

	GLM		GBM		RF		Maxent	
	AUC	TSS	AUC	TSS	AUC	TSS	AUC	TSS
General model	0.661 (0.054)	0.307 (0.058)	0.786 (0.069)	0.439 (0.139)	0.821 (0.043)	0.52 (0.077)	0.784 (0.055)	0.478 (0.082)
*O. m. ferrugineus*	0.897 (0.032)	0.786 (0.054)	0.934 (0.041)	0.797 (0.093)	0.964 (0.025)	0.896 (0.059)	0.976 (0.009)	0.919 (0.048)
*O. m. manul*	0.832 (0.051)	0.569 (0.099)	0.867 (0.068)	0.568 (0.156)	0.85 (0.035)	0.588 (0.072)	0.867 (0.039)	0.615 (0.066)
*O. m. nigripectus*	0.897 (0.051)	0.307 (0.058)	0.8 (0.12)	0.439 (0.139)	0.84 (0.086)	0.52 (0.077)	0.905 (0.027)	0.478 (0.082)

## 4 DISCUSSION

The Pallas's cat is a rare species with an extensive range across the cold steppes of Eurasia (Ross et al., 2020). Presence in such a broad range boosts the likelihood of the existence of different clades in this species. Previous studies have shown that the Pallas's cat has a close phylogenetic relationship with the genus *Prionailurus*, that is, leopard cats, both diverged from their common ancestor roughly 5.9 million years ago (O'Brien & Johnson, 2007). Today, it is known as the only species of the genus *Otocolobus*. Although there is no explicit agreement on the Pallas's cat clades due to the lack of genetic studies (Kitchener et al., 2017), a cluster analysis on climatic variables allowed us to recognize three different clades in this species. Interestingly, the geographic distribution of the identified clusters was consistent with a proposed (Kitchener et al., 2017), yet not genetically confirmed, divergence in the Pallas's cat populations. The distribution of all clades includes a combination of mountain‐steppe areas; however, they show a distinct habitat selection pattern. Evaluating such ecological differences between clades requires an integrated phylogenetically informed SDM analysis. Thus, in the present study, the habitat suitability of the Pallas's cat, as an indicator of the Eurasian cold steppes, was assessed at both species and clade levels.

**FIGURE 5:**
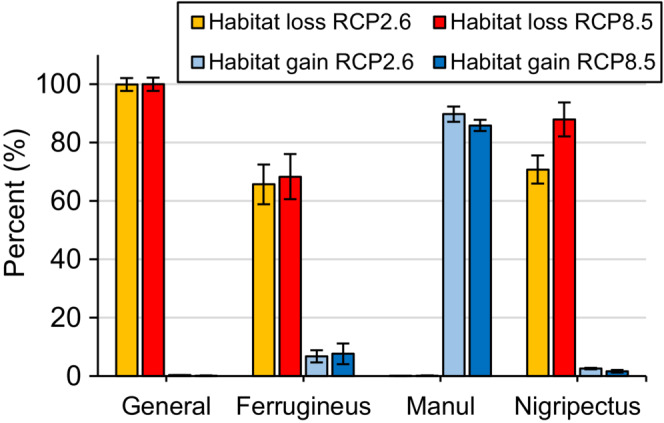
Univariate density plot of environmental variables depicted for each clade of Pallas's cat. For variables description, see Table 1.

As we expected, the AUC and TSS of the models increased significantly with the separation of modeling at the clade level, so that for all models, AUC was more than 0.9, and TSS was more than 0.8. Comparing clades' SDM showed that the highest AUC and TSS were calculated for the Ferrugineus. In terms of climatic variables, temperature seasonality and annual mean temperature were recognized as the most important variables affecting this clade. The univariate comparison of all explanatory variables (Figure 5) indicated that Ferrugineus occupies a narrow and different niche width given these two climatic variables, which both indicate climatic variability of the region. This pattern reveals the specialized habitat use of this species, which has been highlighted by the high value of the AUC and TSS of its model. Being limited to a narrow gradient of environmental conditions, specialist taxa are more predictable and more distinguishable, that is, high values of AUC and TSS of their SDMs, due to the high distinctiveness between their occurrence points and background space (Stolar & Nielsen, 2015). Generally, being a habitat specialist species is the most likely explanation for the low density of Pallas's cats worldwide (Ross, Barashkova, et al., 2019; Ross, Moqanaki, et al., 2019; Ross et al., 2020). However, higher availability of suitable habitats, and most importantly, abundant prey guarantee higher density of the local populations of the species (Anile et al., 2021). Generally, the univariate niche comparison (Figure 5) of all Pallas's cat clades showed that niche occupation was mostly distinctive across temperature‐wise gradients. For example, the distribution of the Manul encompasses a wide range of temperature changes. This is consistent with findings of Munkhtsog et al. (2004), Ross, Barashkova, et al. (2019) and Ross, Moqanaki, et al. (2019) which showed that this clade can tolerate temperature changes ranging from +48°C in summer to −53°C in winter.

Previous studies on Pallas's cat in Iran (the western border of the species global distribution range) show that this species uses a wide range of habitat types (Farhadinia et al., 2016) including high mountain steppes, dry grassland, and temperate shrub lands (Moqanaki et al., 2019). This pattern has been highlighted in the clades' density plot in which the greatest density of the Ferrugineus occurrence points was seen in regions close to both sparse vegetation and denser vegetation, that is, the mixture of grass/scrub/shrub lands. For all clades, we also found a tendency to use areas close to croplands. This is coincident with findings of Greco et al. (2022) where they found Pallas's cat extensively use herbaceous and croplands, and areas with a higher volume of livestock farming.

For Nigripectus, the slope was found one the most critical factor in its habitat use pattern. These results corroborate the findings of a great deal of the previous work; for instance, reports of the species' presence in Tibet have shown altitudes above 5000 m (Chanchani, 2008). For this clade, the altitudes of 5593 and 5050 m were also recorded by Werhahn et al. (2018) and Fox and Dorji (2007), respectively. This pattern differs from the habitat use of the Ferrugineus in Iran where the highest altitude is recorded about at 2500 m (Farhadinia et al., 2016). From a topographic point of view, and contrary to the other two clades, the Manul prefers areas with lower height and topographic diversity, which is likely related to snow depth in high terrains. Snow depth is a limiting factor for Pallas's cat, particularly in the northern part of its distribution, such as Russia and Kyrgyzstan (Barashkova et al., 2017). Snow cover with a depth of 15–20 cm is a significant ecological constraint for the Pallas's cat (Sunquist & Sunquist, 2002). Nevertheless, at a finer scale, the Manul prefers areas with steeper slopes and natural vegetation compared to the local surroundings (Greco et al., 2022).

Ecological niche differences between clades may also stem from their different interactions with other species (Meier et al., 2010; Morales‐Castilla et al., 2017; Pearman et al., 2010). For example, according to our findings, the presence of pika as a preferred prey of the Pallas's cat has a significant impact on the habitat suitability of the Ferrugineus and Nigripectus clades. This also accords with other observations of the Pallas's cat feeding ecology in Iran, showing the wide use of Pika by the species (Adibi et al., 2018). Records of the Pallas's cat from the Tibetan Plateau, where the Nigripectus is spread, also show overlap with *Ochotona curzoniae* (one of the pika species used in our study) (Fox & Dorji, 2007; Webb et al., 2014). Nonetheless, we found that the presence of pika is not an important variable affecting Manul habitat use. Likely, due to the significant impact of temperature on the distribution and habitat selection of pika, these species cannot survive in temperatures above +37°C (Sahneh et al., 2014), while as we indicated earlier, the Manul can tolerate extreme temperatures up to +48°C. The study by Ross et al. (2016) indicated that Pallas's cats are facultative specialist predators in their use of pika, meaning that they can change their diet in response to the availability of this prey species. This feeding behavior of changing preferable prey under different environmental conditions is comparable to the feeding behavior of the Canadian lynx (*Lynx canadensis*) from its preferred prey, Snowshoe hares (*Lepus americanus*) in similar harsh environments (O'Donoghue et al., 1998). Due to the development of local adaptation, clades may respond differently to various environmental conditions (Fournier‐Level et al., 2011). Therefore, one of the advantages of performing within‐taxon SDM analysis could be the possibility of unveiling different biological interactions at the available local conditions.

The considerable differences in the ecological niche of the three Pallas's cat clades significantly affect predicting the impact of climate change on their distribution. Several studies have found that closely related species are likely to have comparable responses to climate change (Ahmadi et al., 2019; Peterson et al., 1999); nonetheless, some research highlighted different responses among closely related taxa (Knouft et al., 2006; Maguire et al., 2018; Pearman et al., 2010; Prasad & Potter, 2017). Our research indicates that the Ferrugineus and Nigripectus clades, due to a great extent of range loss, are probably more threatened by climate change than Manul for which range gain was projected to be greater than range loss. This finding is contrary to the results of Ye et al. (2018), who found that for Pallas's cat of the Chinese part of the Altai Mountains, which is equivalent to the distribution range of the Manul, range loss will be greater than range gain by 2050. This disagreement might be due to the difference in the geographical extent of the two studies, while they only considered a limited part of the species range our study encompasses its entire range. Furthermore, we applied the multivariate environmental similarity surfaces (MESS) analysis in our climate change projection. By doing so, we removed novel habitats that are prone to extrapolation because of their dissimilar climatic conditions to the species' current range (Elith et al., 2010). It is worth mentioning that the extrapolation to inaccessible areas was more evident for the clades Ferrugineus and Nigripectus (Figure 4), which interestingly showed higher specialization across the gradient of the environmental variables (Figure 3). This may bring to mind that habitat specialist species are probably more prone to extrapolation in climate change projections, a pattern that requires further investigation in future studies.

In our study, the distinct response of clades to climate change was buried in the general climate change model of the species in which Pallas's cat was projected to lose most of its habitat across its entire range. Accordingly, our findings are exceedingly consistent with the results of Pearman et al. (2010) and Prasad and Potter (2017) in supporting the idea that projections for clades and subclades can be different in showing the effects of climate change. Consequently, models that combine different within‐taxa structures, due to fitting smoothed response curves of the explanatory variables, are prone to model generalization in climate change projections (Pearman et al., 2010). The integration of intraspecific genetic variation and species distribution models in generating a more precise and robust representation of species distribution has also been highlighted in similar studies (Ahmadi et al., 2018; Milanesi et al., 2018).

It is worth bearing in mind that the observed differences in the ecological niche of the three proposed Pallas cat's clades are contrary to the presumption of Kitchener et al. (2017), who based on only the geographic distribution assumed Ferrugineus and Manul clades as the same group and suggested Manul and Nigripectus as the only probable clades. This issue will require further detailed studies on the phylogenetic relationship and population genetics of the species, a shortcoming that we acknowledge in our study. Unveiling gene flow and habitat connectivity patterns are essential for priority‐setting conservation planning to ensure the long‐term persistence of the species. Since one of the most crucial threats to Pallas's cat throughout its distribution is dominant competitors and predators such as large raptors, red fox (*Vulpes vulpes*), gray wolf (*Canis lupus*), and herding dogs (Barashkova et al., 2019; Farhadinia et al., 2016; Ross et al., 2020), it is essential to examine their influence in evaluating habitat selection of the species, especially at fine‐scale levels.

Another important issue in assessing species distribution is the taxonomic and observational bias caused by imperfect detection of the target species (Araújo & Guisan, 2006). Similar is the case when the target species is rare (Zhang et al., 2020) or data on the species come from different resources, such as museums or obtaining data from the GBIF dataset (Troudet et al., 2017). In this situation, ensuring that the collected data correctly represent the actual distribution of the species (Guillera‐Arroita et al., 2015) and reducing biased recognition of the species taxonomy (Rocchini et al., 2011) improve the results of an SDM analysis. Regardless of any shortcomings, our approach shed light on the distribution of different clades of Pallas's cat throughout the cold steppes of Eurasia. The divergent habitat use of the species supported our hypothesis of the existence of three different clades in this species. This pattern bears in mind that a global evaluation of the species' status in the Red List of IUCN might neglect the regional/national specific status that are necessary for the conservation planning of the species. More importantly, since the rate of the loss of phylogenetic diversity is higher than that of species diversity (Buerki et al., 2015), focus on within‐taxon structures might be more efficient in determining extinction risk and implementing conservation measures of widespread species. Although the IUCN's criteria of extinction risk have increasingly been adopted at national levels (Miller et al., 2007), our finding on the global distribution of the Pallas's cat brings the role of trans‐boundary conservation partnership into a new focus.

## 5 CONCLUSIONS

In this research, we evaluated the distribution of Pallas's cat as a widespread but isolated species regarding the distinct genetic structures within the species. Based on a set of findings, the used approach yielded well‐founded results. First, within‐species SDMs had higher predictive performance (AUC and TSS) than the general niche model of the species. Second, this was well depicted in the response curves of the models as well as the univariate density plots where the smoothed gradient of the general model buried the niche occupation of the clades. Subsequently, it was possible to assess the niche partition of the clades more efficiently. Third, the negative effect of imbalanced‐biased data sampling of the species over its entire global distribution in the modeling approach was reduced. Accordingly, we extended our clades modeling attitude to climate change projections, and fourth, this procedure allowed us to identify clades that are more sensitive to future climate change. Altogether, the present study shows that for species with widespread distribution ranges but isolated populations, the within‐taxon modeling approach can more effectively address local adaptation patterns and illustrate cryptic details of the species distribution.

## AUTHOR CONTRIBUTIONS

**Niloufar Lorestani:** Conceptualization (equal); formal analysis (equal); investigation (equal); methodology (equal); writing – original draft (equal); writing – review and editing (equal). **Mahmoud‐Reza Hemami:** Conceptualization (equal); methodology (equal); writing – original draft (supporting); writing – review and editing (equal). **Azita Rezvani:** Investigation (equal); writing – original draft (equal); writing – review and editing (equal). **Mohsen Ahmadi:** Conceptualization (lead); formal analysis (lead); investigation (equal); methodology (equal); writing – original draft (equal); writing – review and editing (lead).

## CONFLICT OF INTEREST

The authors declare no conflict of interest.

## Supporting information


**Appendix S1.** Supporting Information

## Data Availability

All references that were used to confirm the GBIF data are provided in Supplementary Materials S1.
