# Supplementary material for: Ecological niche models reveal divergent habitat use of Pallas's cat in the Eurasian cold steppes
Source: Ecol Evol. 2022 Dec 14;12(12):e9624. doi: 10.1002/ece3.9624 (PMC9750817; doi:10.1002/ece3.9624)
Supplement: Supplementary file 1 — Appendix S1. Supporting Information [file ECE3-12-e9624-s001.docx]

**Appendix S1**

**Table S1.** The details of the literature review that used to confirmed GBIFs' points.

| **Category** | **Country** | **Number of points** | **Sources** |
| --- | --- | --- | --- |
| **South-western** | Iran | 36 | (Moqanaki et al., 2019),(Farhadinia et al., 2016), (Aghili et al., 2008),(Chalani et al., 2008), (Dibadj et al., 2018), (Karami et al., 2016), Department of Environment of Iran (DOE), (Ross et al., 2020), (Aghili-2004- Report to DOE), (Lanz et al., 2019), (Adibi et al., 2018), (Yusefi et al., 2019), http://www.iew.ir, http://wildcats.wildlifemonitoring.ru , (CatNews: http://www.catsg.org/), Field observations, Pallas' Cat photographed |
|  | Turkmenistan | 1 | (Moqanaki et al., 2019), http://wildcats.wildlifemonitoring.ru |
|  | Azerbaijan | 1 | http://wildcats.wildlifemonitoring.ru |
| **Himalayas and China** | Afghanistan | 3 | (Habibi k, 2004), (Moqanaki et al., 2019), http://wildcats.wildlifemonitoring.ru |
|  | Pakistan | 2 | (Hameed & Nawaz, 2014), http://wildcats.wildlifemonitoring.ru |
|  | India | 3 | (Chanchani, 2008), (Pal et al., 2019), (Dhendup et al., 2019), http://wildcats.wildlifemonitoring.ru |
|  | Nepal | 1 | (Werhahn et al., 2018), (Dhendup et al., 2019) http://wildcats.wildlifemonitoring.ru |
|  | Bhutan | 1 | (Thinley, 2013), (Ross et al., 2020), <http://wildcats.wildlifemonitoring.ru>, |
|  | China | 10 | (Dhendup et al., 2019), (Webb et al., 2014), (Fox & Dorji, 2007), (Ross et al., 2020), (Lanz et al., 2019), (Mallon, 2002), <http://wildcats.wildlifemonitoring.ru>, |
| **Central Asia** | Mongolia | 16 | (Lanz et al., 2019), (Barashkova et al., 2019), (Jutzeler et al., 2010), (Faucher 2018, Available at https://www.inaturalist.org/observations/16891147), <http://wildcats.wildlifemonitoring.ru>, |
|  | Kazakhstan | 18 | (Barashkova et al., 2019), (Lanz et al., 2019) <http://wildcats.wildlifemonitoring.ru>, |
|  | Kyrgyzstan | 4 | (Barashkova et al., 2019), (Lanz et al., 2019),  <http://wildcats.wildlifemonitoring.ru>, |
|  | Russia | 41 | (Barashkova et al., 2019), (Lanz et al., 2019), (Barashkova et al., 2017), <http://wildcats.wildlifemonitoring.ru>, |


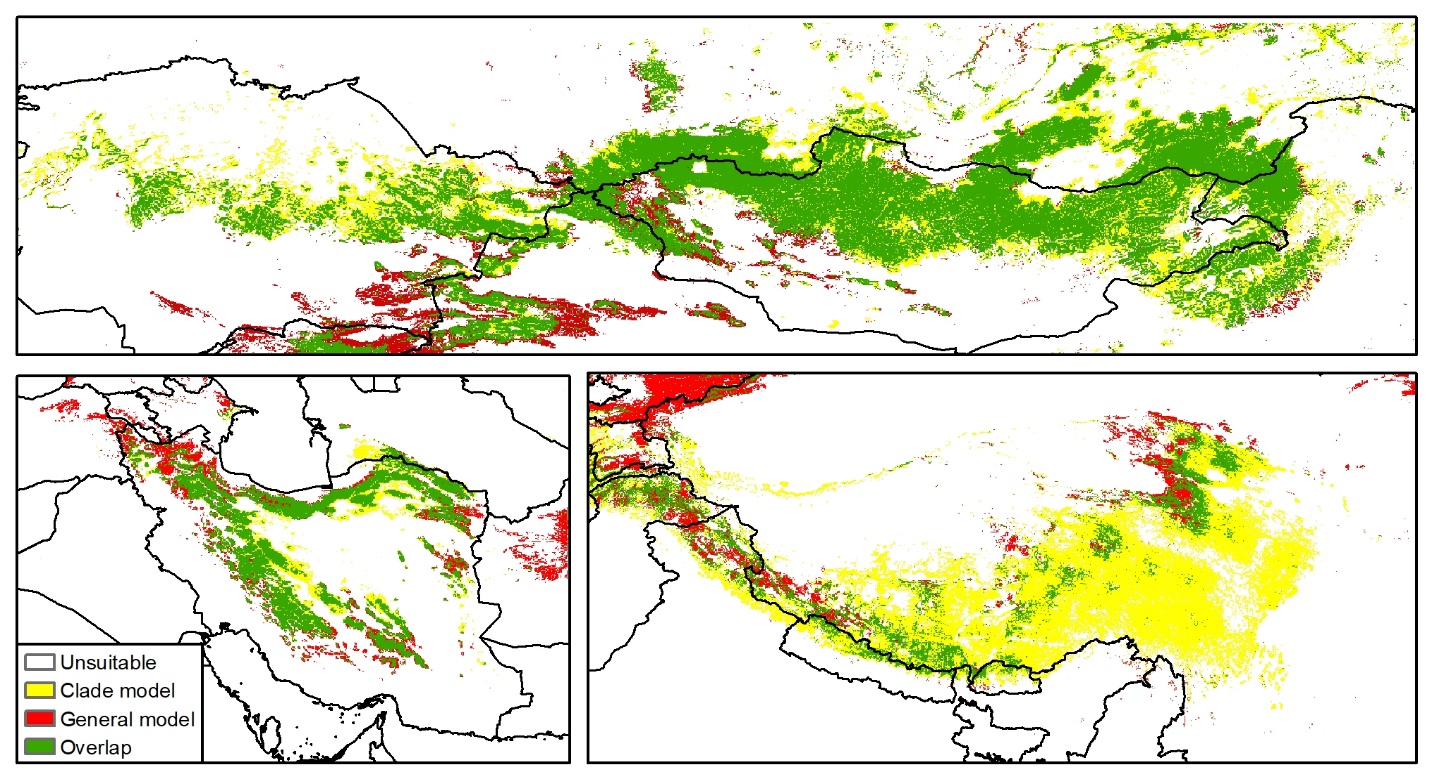


**Figure S1.** The geographic distribution of the binary habitat suitability maps of the Pallas’s cat’s clades and the spatial overlap between them. To generate binary maps a threshold of 10th percentile training presence was used.

**Pika habitat suitability**

We used 6 species of Pika to incorporate preferred prey availability in the habitat suitability modeling of Pallas's cat, including (northern pika *Ochotona hyperborea*, plateau pika *Ochotona curzoniae*, large-eared pika *Ochotona macrotis*, Pallas's pika *Ochotona pallasi*, Afghan pika *Ochotona rufescens*, and Moupin pika *Ochotona thibetana*). We select these pika species according to their spatial affinity with the Pallas's cat and categorized them in three classes:

(ⅰ) Central Asia, where the distribution of two pika species, northern pika and Pallas's pika, has overlap with *O. m. manul*. To model habitat suitability of these pika species we obtained 100 occurrence points from GBIF.

(ⅱ) Southwest of the study area, where the distribution of Afghan pika is overlapped with the distribution of *O. m. ferrugineus.* We used the presence points of the species obtained from GBIF as well as Atlas of Iranian Mammals (Karami et al. 2016) and checked with (Khakisahne et al. 2013) (n=88).

(ⅲ) Himalayas and China, where the distribution of three pika species, large-eared pika, plateau pika, and Moupin pika, has overlap with *O. m. nigripectus*. For the habitat suitability of these pika species we downloaded 115 occurrence points from the GBIF.

After filtering out duplicates, 303 occurrence records remained. We modeled the habitat suitability of pika with the same selected layers used for Pallas's cat’s habitat suitability model. Modeling was performed by using the package biomod2 in the R environment, based on an ensemble model of four algorithms: GLM, GBM, RF, and Maxent.


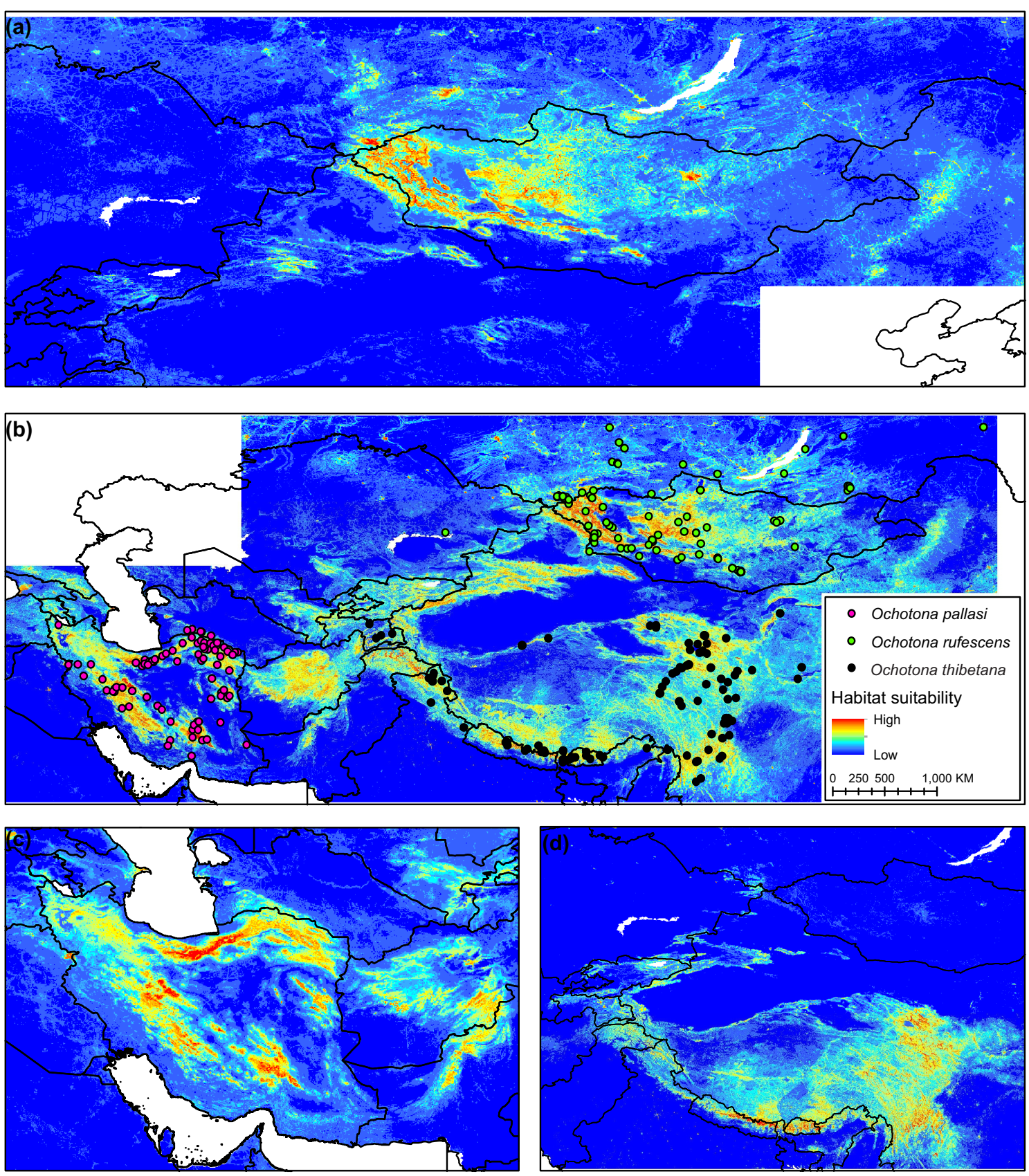


**Figure S2.** Habitat suitability map of Pika derived based on the general model (pooled set of the occurrence points of the subspecies) and individual species models.

**Appendix S.1 references**

Adibi, M. A., Shirazi, M. R., & Moqanaki, E. (2018). A Pallas ’ s cat roadkill in Iran. Cat News, 68.

Aghili, A, Masoud, R., Murdoch, J. D., & Mallon, D. P. (2008). First Record of Pallas’s Cat in Northwest Iran. Cat News, 49, 8–9.

Barashkova, A. N., Kirilyuk, V. E., & Smelansky, I. E. (2017). Significance of Protected Areas for the Pallas’s cat (Otocolobus manul: Felidae) conservation in Russia. Nature Conservation Research, 2(1), 113–124. https://doi.org/10.24189/ncr.2017.019

Barashkova, A. N., Smelansky, I. E., Kirilyuk, V. E., Sergey, N., Anastazia, A., Uulu, K. Z., Koshkin, M. A., Nasanbat, B., Baatargal, O., Grachev, A., & Lissovsky, A. (2019). Distribution and status of the manul in Central Asia and adjacent areas. Cat News, 14–23(13).

Chalani, M., Ghoddousi, A., Ghadirian, T., & Goljani, R. (2008). First Pallas’s Cat Photo-trapped in Khojir National Park, Iran. Cat News, 49, 7.

Chanchani, P. (2008). Sighting of a Manul or Pallas Cat in North Sikkim, India. Cat News, 48, 18–19.

Dhendup, T., Shrestha, B., Mahar, N., Kolipaka, S., RamRegmi, G., & Jackson, R. (2019). Distribution and status of the manul in the Himalayas and China. Cat News Special Issue:Pallas’s Cat Status Review & Conservation Strategy, 31–36.

Dibadj, P., Jafari, B., Nejat, F., Qashqaei, A. T., & Ross, S. (2018). Maternal habitat use of Juniperus excelsa woodland by Pallas’s cat Otocolobus manul in Iran. Zoology and Ecology, 28(4), 421–424. https://doi.org/10.1080/21658005.2018.1520722

Farhadinia, M. S., Moqanaki, E. M., & Adibi, M. A. (2016). Baseline information and status assessment of the Pal- las’s cat in Iran. Cat News, 10, 38–42.

Fox, J. L., & Dorji, T. (2007). High elevation record for occurrence of manul or Pallas cat on the northwestern Tibetan Plateau, China. Cat News, 46, 35.

Habibi k. (2004). Mammals of Afghanistan. In Zoo Outreach Organization.

Hameed, S., & Nawaz, M. A. (2014). Pallas’s cat photographed in Qurumber National Park, Gilgit-Baltistan, Pakistan. Cat News, 60 (April).

Jutzeler, E. V. A., Yan, X. I. E., & Vogt, K. (2010). The smaller felids of China Pallas ’ s cat. Cat News Special Issue 5, 37–39.

Karami, M., Ghadirian, T., & Faizolahi, K. (2016). The Atlas of Mammals of Iran. Department of Environment and University of Tehran.

Lanz, T., Breitenmoser, C., Barclay, D., Nygren, E., Samelius, G., & Breitenmoser, U. (2019). Why care about Otocolobus manul? Cat News Special, 13, 5–8.

Mallon, D. (2002). Manul sighting in Qinghai, China. Cat News, 36, 18.

Moqanaki, E., Jahed, N., Malkhasyan, A., Askerov, E., Farhadinia, M., Kabir, M., Adibi, M. A., Din, J., Joolaee, L., Chahartaghi, N. R., & Ostrowski, S. (2019). Distribution and status of the Pallas’s cat in the south-west part of its range. Cat News Special Issue:Pallas’s Cat Status Review & Conservation Strategy, 13, 24–30.

Pal, R., Bhattacharya, T., & Sambandam, S. (2019). First record of Pallas’s cat in Uttarakhand, Nelang valley, Gangotri National Park, India. June.

Ross, S., Barashkova, A. N., Dhendup, T., Munkhtsog, B., Smelansky, I., Barclay, D., & Moqanaki, E. (2020). Otocolobus manul. The IUCN Red List of Threatened Species 2020, e.T15640A1. https://dx.doi.org/10.2305/IUCN.UK.2020-2.RLTS.T15640A162537635.en

Thinley, P. (2013). First photographic evidence of Pallas’s cat in Jigme Dorji National Park, Bhutan. Cat News, 58, 27–28.

Webb, R., Pain, D., McNiven, D., & Francis, S. (2014). Pallas’s cat in disturbed habitat on the Tibetan Plateau. Cat News, Spring(60), 27–28.

Werhahn, G., Kusi, N., Karmacharya, D., Sherchan, A., Manandhar, P., Manandhar, S., Bhatta, T., Joshi, J., Bhattarai, S., Sharma, A., Kaden, J., Ghazali, M., & Senn, H. (2018). Eurasian lynx and Pallas’s cat in Dolpa district of Nepal: genetics, distribution and diet. Cat News, 67, 34–36.

Yusefi, G., Faizolahi, K., Darvish, J., Safi, K., & Brito, J. (2019). The species diversity, distribution, and conservation status of the terrestrial mammals of Iran. Journal of Mammalogy, 100 (1), 55–71. https://doi.org/10.1093/jmammal/gyz002
